# Salvianolic acid B attenuates tubulointerstitial fibrosis by inhibiting EZH2 to regulate the PTEN/Akt pathway

**DOI:** 10.1080/13880209.2022.2148169

**Published:** 2022-12-16

**Authors:** Pinglan Lin, Furong Qiu, Ming Wu, Lin Xu, Di Huang, Chen Wang, Xuejun Yang, Chaoyang Ye

**Affiliations:** a Department of Nephrology, Shuguang Hospital Affiliated to Shanghai University of Traditional Chinese Medicine; b TCM Institute of Kidney Disease, Shanghai University of Traditional Chinese Medicine, Shanghai, P. R. China; c Key Laboratory of Liver and Kidney Diseases (Shanghai University of Traditional Chinese Medicine), Ministry of Education, Shanghai, P. R. China; d Laboratory of Clinical Pharmacokinetics, Shuguang Hospital Affiliated to Shanghai University of Traditional Chinese Medicine, Shanghai, P. R. China

**Keywords:** Renal fibrosis, chronic kidney diseases, ureteral unilateral obstruction, Aristolochic acid nephropathy, transforming growth factor-β

## Abstract

**Context:**

Salvianolic acid B (SAB) can alleviate renal fibrosis and improve the renal function.

**Objective:**

To investigate the effect of SAB on renal tubulointerstitial fibrosis and explore its underlying mechanisms.

**Materials and methods:**

Male C57 mice were subjected to unilateral ureteric obstruction (UUO) and aristolochic acid nephropathy (AAN) for renal fibrosis indication. Vehicle or SAB (10 mg/kg/d, i.p.) were given consecutively for 2 weeks in UUO mice while 4 weeks in AAN mice. The serum creatinine (Scr) and blood urine nitrogen (BUN) were measured. Masson’s trichrome staining and the fibrotic markers (FN and α-SMA) were used to evaluate renal fibrosis. NRK-49F cells exposed to 2.5 ng/mL TGF-β were treated with SAB in the presence or absence of 20 μM 3-DZNep, an inhibitor of EZH2. The protein expression of EZH2, H3k27me3 and PTEN/Akt signaling pathway in renal tissue and NRK-49F cells were measured by Western blots.

**Results:**

SAB significantly improved the levels of Scr by 24.3% and BUN by 35.7% in AAN mice. SAB reduced renal interstitial collagen deposition by 34.7% in UUO mice and 72.8% in AAN mice. Both *in vivo* and *in vitro* studies demonstrated that SAB suppressed the expression of FN and α-SMA, increased PTEN and decreased the phosphorylation of Akt, which were correlated with the down-regulation of EZH2 and H3k27me3. The inhibition of EZH2 attenuated the anti-fibrotic effects of SAB in NRK-49Fs.

**Conclusion:**

SAB might have therapeutic potential on renal fibrosis of CKD through inhibiting EZH2, which encourages further clinical trials.

## Introduction

The prevalence of chronic kidney disease (CKD) is over 10.8% of the human population worldwide with high mortality and disability rates, and remains a major public health burden (Webster et al. 2017). Renal fibrosis is characterized by activation of renal interstitial fibroblasts, renal epithelial mesenchymal transition (EMT) and excessive accumulation of extracellular matrix (ECM) proteins, which leads to functional deterioration and renal structure destruction with the repeated injury of renal tubulars or glomerulus (Liu et al. 2018). If not prevented or reversed, fibrosis will be the common pathologic process that drives progression to end-stage renal disease (ERSD) (Humphreys 2018). Angiotensin-converting enzyme inhibitor and angiotensin receptor blocker (ARB) are the current drugs used to ameliorate renal fibrosis (Nastase et al. 2018). Due to the limitations of western medicine and the unspecified pathological mechanism in curing or postponement of renal fibrosis, studies are focused on Chinese herb medicine treatment and its underlying mechanism (Shao et al. 2021).

Transforming growth factor (TGF-β)/Smad3 signaling pathways play a predominant role in renal fibrosis. TGF-β1 is the paracrine factor released by epithelial cells binding to TGF-β receptor II induces its activation and then driving expression of multiple genes associated with renal injury and fibrosis such as α-smooth muscle actin (α-SMA) and fibronectin (FN) (Ruiz-Ortega et al. 2020). In renal fibrosis and diabetic nephropathy model, injury to the kidney or cultured human kidney proximal tubular cells results in the down-regulation of phosphatase and tensin homolog (PTEN), which is a protein tyrosine phosphatase that dephosphorylates the protein kinase B (Akt) (An et al. 2022). Activation of Akt signaling can inhibit profibrotic signaling pathways, renal inflammation response and epithelial cell trans-differentiation, subsequently alleviates renal fibrosis (Lan and Du 2015).

Enhancer of zeste homolog-2 (EZH2) is a methyltransferase that induces histone H3 lysine 27 trimethylation (H3K27me3) to regulate the gene transcription in fibrogenesis (Zhou et al. 2016). Studies demonstrated that EZH2 expression was upregulated in renal fibrosis induced by the murine model of UUO or TGF-β stimulated, which contributed tubular epithelial EMT and fibrosis by downregulating expression of PTEN, thus activating multiple profibrotic signaling pathways (Zhou et al. 2018).

Salvianolic acid B (SAB) is a water-soluble compound in *Salvia miltiorrhiza* Bunge (Libiatae) (known as ‘Danshen’ in Chinese), which is widely used in treating cardiovascular (Wu et al. 2020), renal (Lin et al. 2019) and hepatic disease with antioxidative and cell protective properties (Liu et al. 2021). Recently, many studies demonstrated that SAB also had a good effect on the alleviation of renal fibrosis by reducing inflammation and oxidative stress and attenuating the EMT within fibroblasts (Ma et al. 2019). However, the potential mechanisms of action remain to be further elucidated. In this study, we investigate whether SAB could improve renal interstitial fibrosis (RIF) by inhibiting EZH2 to regulate the PTEN/Akt signaling pathway.

## Materials and methods

### Animal studies protocol

Male c57 mice (SPF grade) weighting between 20 and 25 g were purchased from Shanghai SLAC Laboratory Animal Co., Ltd. The animals were housed in the animal Centre of Shanghai University of Traditional Chinese Medicine according to local regulations and guidelines. Animal experiments described herein were endorsed by the animal experimentation ethics committee of Shanghai University of Traditional Chinese Medicine (PZSHUTCM190920019).

For unilateral ureter obstructed (UUO) mouse model, 8-week-old mice were anaesthetized with sodium pentobarbital (SP) (8 mg/kg, i.p.), and then a left flank incision was made to expose the left ureter which was ligated with 4–0 silk sutures. The same operation was performed in sham mice except the ligation of ureter. Twenty-eight mice were randomly divided into four groups: (I) sham + Vehicle (saline) group (*n* = 7), (II) UUO + Vehicle (saline) group (*n* = 7), (III) sham + SAB (10 mg/kg) group (*n* = 7), and (IV) UUO + SAB (10 mg/kg) group (*n* = 7). One day after the surgery, mice were treated with saline or 10 mg/kg SAB daily by intraperitoneally (i.p.) injection for 13 consecutive days. 14 days after the operation, mice were euthanized with SP (8 mg/kg, i.p.) and kidney tissues were collected for histopathological and molecular examination.

For the aristolochic acid nephropathy (AAN) mouse model, 8-week-old mice was constructed by intraperitoneally (i.p.) injection of aristolochic acid I (dissolved in saline supplemented with 3%DMSO) at a dose of 3 mg/kg/every 3 days once for 6 consecutive weeks to establish progressive fibrosis. The normal control group was injected with the same volume of 3% DMSO saline over the same 6-week period. Two weeks after the AAI injection, mice were treated with saline or 10 mg/kg SAB daily by intraperitoneally (i.p.) injection for four weeks. Mice were randomly divided into four different groups: (I) Control + Vehicle (saline) group (*n* = 8), (II) AAN + Vehicle (saline) group (*n* = 8), (III) Control + SAB (10 mg/kg) group (*n* = 8), and (IV) AAN + SAB (10 mg/kg) group (*n* = 8). 4 weeks after SAB treatment, mice were euthanized with SP (8 mg/kg, i.p.) and kidney tissues were collected for histopathological and molecular examination. Blood samples were centrifuged at 3500 rpm for 10 min to collect serum. Serum contents of creatinine (Scr) and urea nitrogen (BUN) were detected by automatic biochemical analyser (AU680, Beckman Coulter, USA) in Clinical Laboratory of Shuguang Hospital.

### Cell culture and treatment

NRK-49F cells were purchased from the National Collection of Authenticated Cell Cultures, Chinese Academy of Medical Sciences. The cells were cultured in DMEM/F12 (GNM12400, GENOM, China) medium containing 10% foetal bovine serum (04-001-1ACS, Biological Industries, Israel) and 0.5% penicillin/streptomycin (C0222, Beyotime Biotech, China) in an atmosphere of 5% CO_2_ at 37 °C. NRK-49F cells were seeded in 6-well plates and starved for 12 h before the stimulation with 2.5 ng/mL TGF-β1 (100-21, PeproTech, USA) when cells reached 60–70% confluence. SAB at three concentration (3, 10, 30 μM) or 20 μM 3-DZNep were added after TGF-β challenge. The cells were cultured for 24 h with treatment and protein was collect for Western blot analysis. All experiments were repeated at least three times.

### Histological analysis and immunohistochemical staining

Kidneys were fixed in 4% paraformaldehyde and embedded with paraffin. Tissue sections 4 µm thick were performed to Masson’s trichrome (Masson) staining according to the protocol. For immunohistochemical staining, the 4 μm sections were incubated with 1:200 diluted PTEN rabbit Anti-body (9559S, CST, USA). Images were obtained with the use of a microscope (80i, Nikon, Japan).

### Cell viability assay

Cells were seeded in 96-well plates (5 × 10^3^ cells/well) for 12 h and then incubated with various concentration of SAB at 0, 0.3, 1, 3, 10, 30, 100, 300 μM. A CCK-8 solution (40203ES60, Yeasen, China) was added after 24 h. The OD value at the wavelength of 450 nm was detected using the microplate reader (Cytation 3, Biotek, USA) after incubating for 60 min.

### Western blotting analysis

Protein was extracted from cells or mouse kidneys using RIPA lysis buffer (P0013B, Beyotime Biotech, China). The protein concentration was measured by the BCA protein assay kit, and then dissolved in 5 × SDS-PAGE loading buffer. Samples were separated by 8% SDS-PAGE gels. After electrophoresis, proteins were electro-transferred to a polyvinylidene difluoride membrane (Merck Millipore, Germany), which was incubated in the blocking buffer (5% non-fat milk, 20 mM Tris-HCl, 150 mM NaCl, pH 8.0, and 0.1% Tween 20) for 1 h at room temperature and was followed by incubation with antibodies for fibronectin (ab45688, Abcam, UK), α-SMA (ET1607-53, HUABIO, China), EZH2 (5246, CST, USA), H3K27me3 (9733, CST, USA), PTEN(WL01901, Wanleibio, China), Akt(pan) (4691, CST, USA), Anti-phospho-akt1(Ser473) (ET1607-73, HUABIO, China), GAPDH (6004-1-lg, Proteintech, China) overnight at 4 °C. Binding of the primary antibody was detected by ECL method (180-501 ECL, Tanon, China) using horseradish peroxidase-conjugated secondary antibodies (goat anti-rabbit IgG, A0208 or goat anti-mouse IgG, A0216, Beyotime Biotech, China). The band densities were measured using Quantity One software (Bio-Rad, USA).

### Statistical analysis

Results were presented as mean ± SD. Differences among multiple groups were analyzed by one-way analysis of variance (ANOVA) and comparison between two groups was performed by *t*-test. Statistic software SPSS 26.0 (SPSS Inc., USA) and GraphPad Prism 8.0 software (GraphPad Software, USA) were used. A *p* value <0.05 was considered statistically significant.

## Results

### SAB alleviated renal tubulointerstitial fibrosis

**Figure 1.**
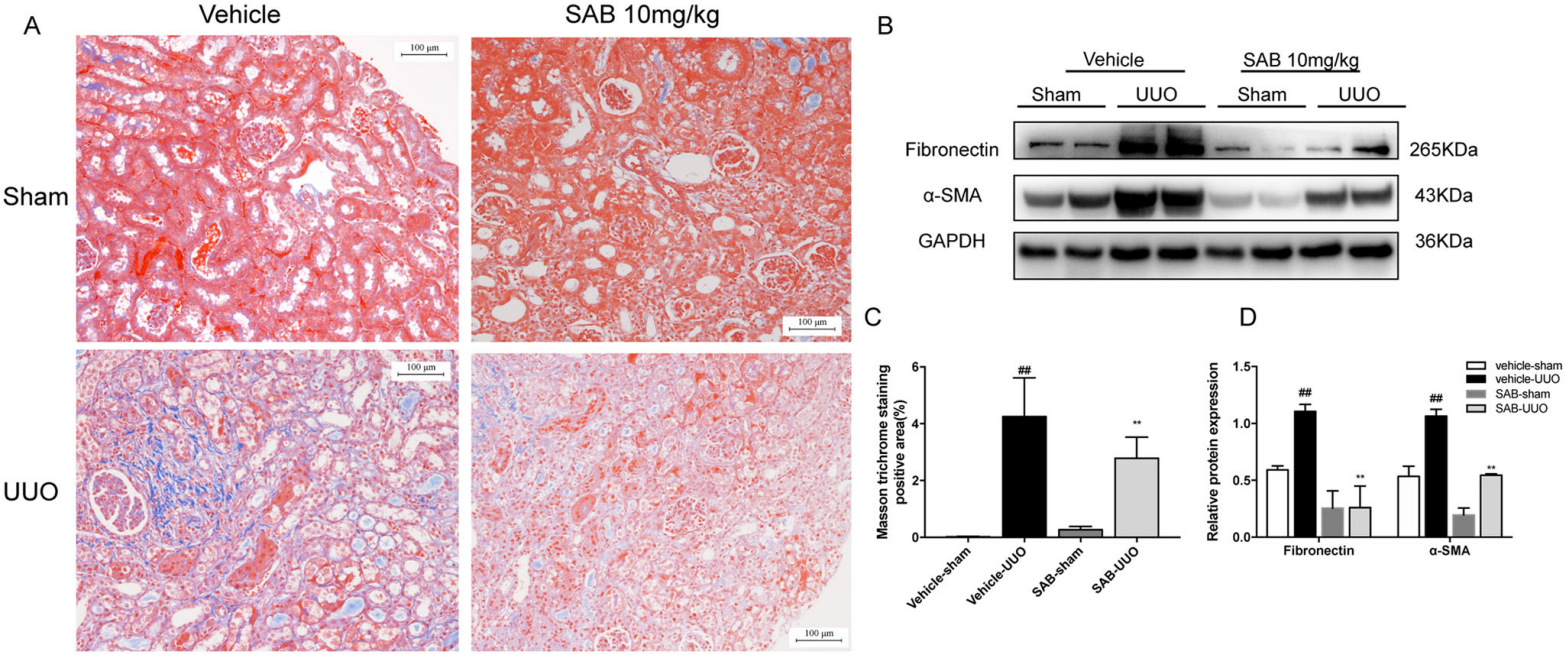
SAB alleviates renal fibrosis in the UUO model of CKD. (A) Renal fibrosis was assessed by Masson’s trichrome staining (magnification ×200, Bar = 100 μm). (B) Immunoblot analysis of FN and α-SMA expression. (C) Semiquantitative result of collagen area (*n* = 6). (D) Western blot quantification of FN and α-SMA levels (*n* = 6). Data represent mean ± SD. ^##^*p* < 0.01 vs. vehicle-sham; **p < 0.01 vs. vehicle-UUO.

**Figure 2.**
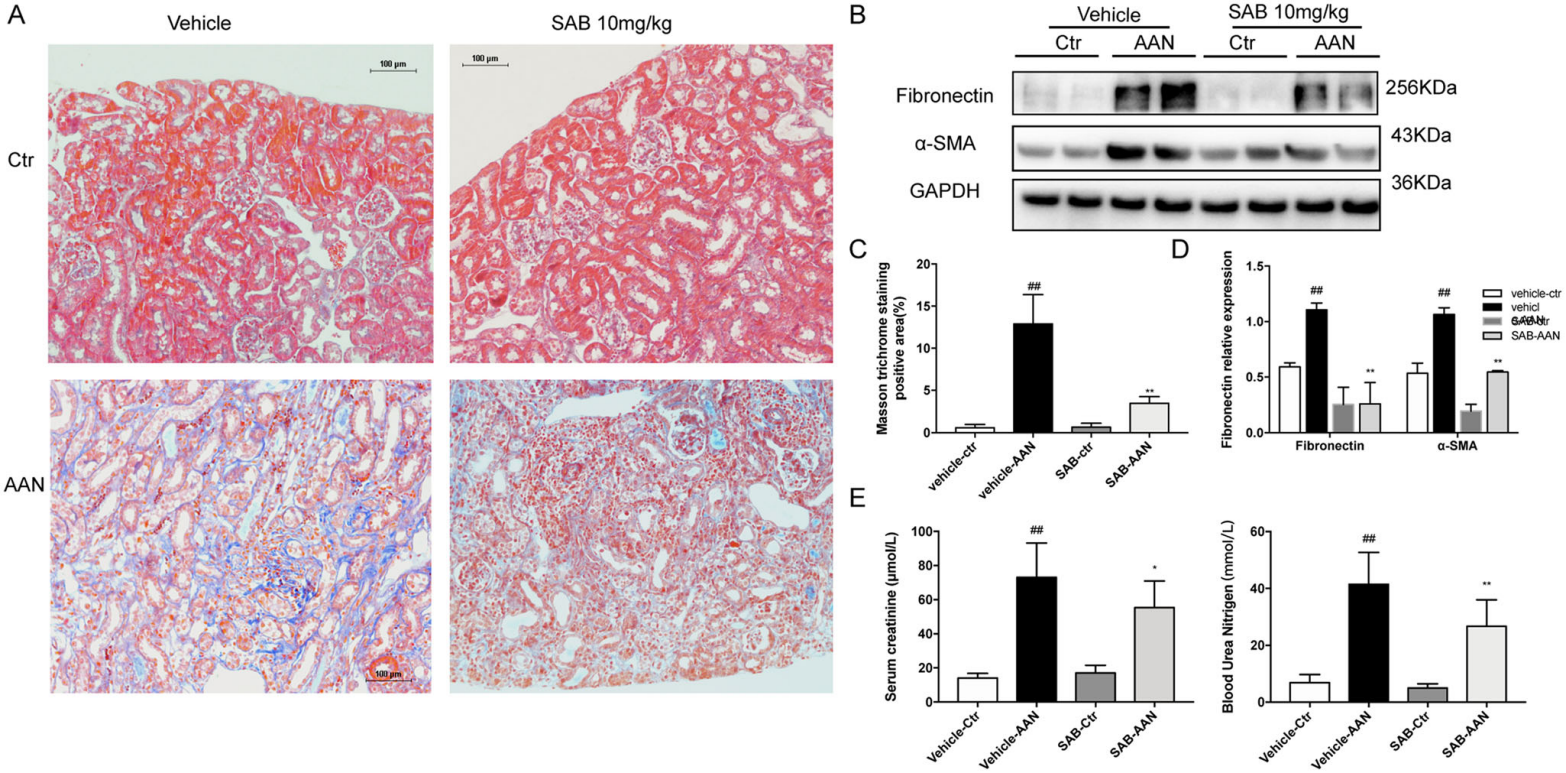
SAB protects against the development of renal fibrosis and renal function in the AAN model of CKD. (A) Renal fibrosis was assessed by Masson’s trichrome staining (magnification ×200, Bar = 100 μm). (B) Immunoblot analysis of FN and αSMA expression. (C) Semiquantitative result of collagen area (*n* = 6). (D) Western blot quantification of FN and α-SMA levels (*n* = 6). (E) The levels of serum creatinine (Scr) and blood urea nitrogen (BUN) were measured. ^##^*p* < 0.01 vs. vehicle-control; *p < 0.05 vs. vehicle-AAN; **p < 0.01 vs. vehicle-AAN.

*In vivo*, inhibition of SAB against renal fibrosis was investigated in UUO and AAN mouse models. Both models lead to the progressive development of interstitial fibrosis in kidneys as assessed by Masson’s trichrome staining, which were attenuated with SAB. (Figures 1(A,C) and 2(A,C)). The protein expression of FN and α-SMA were up-regulated in the kidney of UUO or AAN mouse model compared with the sham or vehicle group. However, treatment with SAB can significantly reduce the expression of these pro-fibrotic proteins of kidney tissues in both mouse models (Figures 1(B,D) and 2(B,D)). Figure 2(E) showed that the Scr and BUN levels in the AAN model group was significantly (*p* < 0.01) higher than that in control group. Four weeks of treatment with SAB significantly reduced the Scr levels by 48.3% (*p* < 0.01) and the BUN levels were 66.7% (*p* < 0.01) lower in the AAN + SAB group than that in the AAN model group.

**Figure 3.**
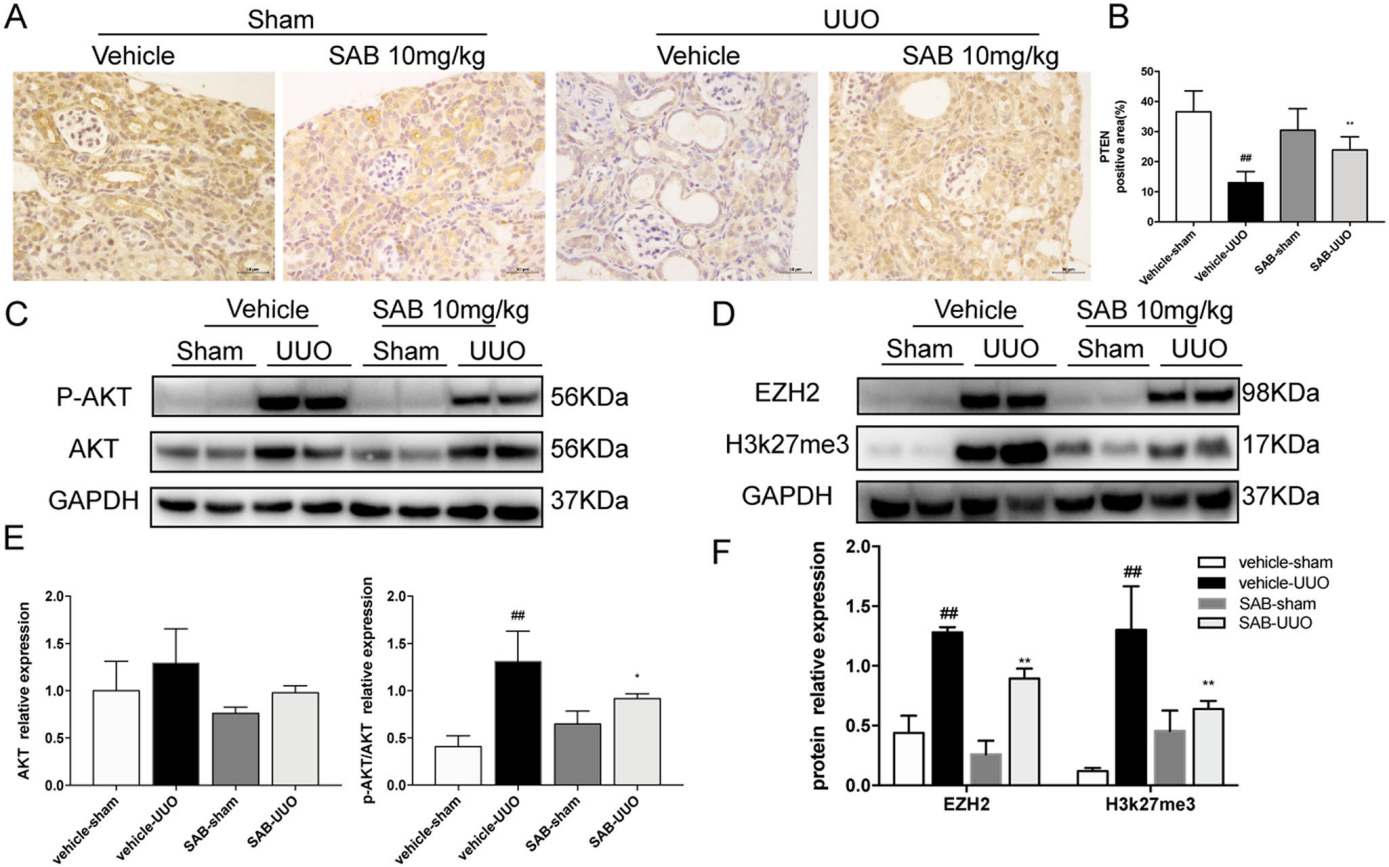
SAB inhibits renal fibrosis through inhibiting EZH2 and reversing the low level of PTEN and phosphorylation of AKT increase in obstructed kidneys. (A) Representative images of PTEN expression detected by IHC (Magnification × 400, Bar = 50 μm). (B) Quantitative analysis of PTEN positive area (*n* = 6). (C) Immunoblot analysis of p-AKT and AKT. (D) Immunoblot analysis of EZH2 and H3k27me3. (E) The ratio of AKT to GAPDH protein and phosphorylated AKT to AKT protein was measured (*n* = 6). (F) The ratio of EZH2, H3k27me3 to GAPDH protein was measured (*n* = 6). ^##^*p* < 0.01 vs. vehicle-sham vs. vehicle-sham; *p < 0.05 vs. vehicle-UUO; **p < 0.01 vs. vehicle-UUO.

**Figure 4.**
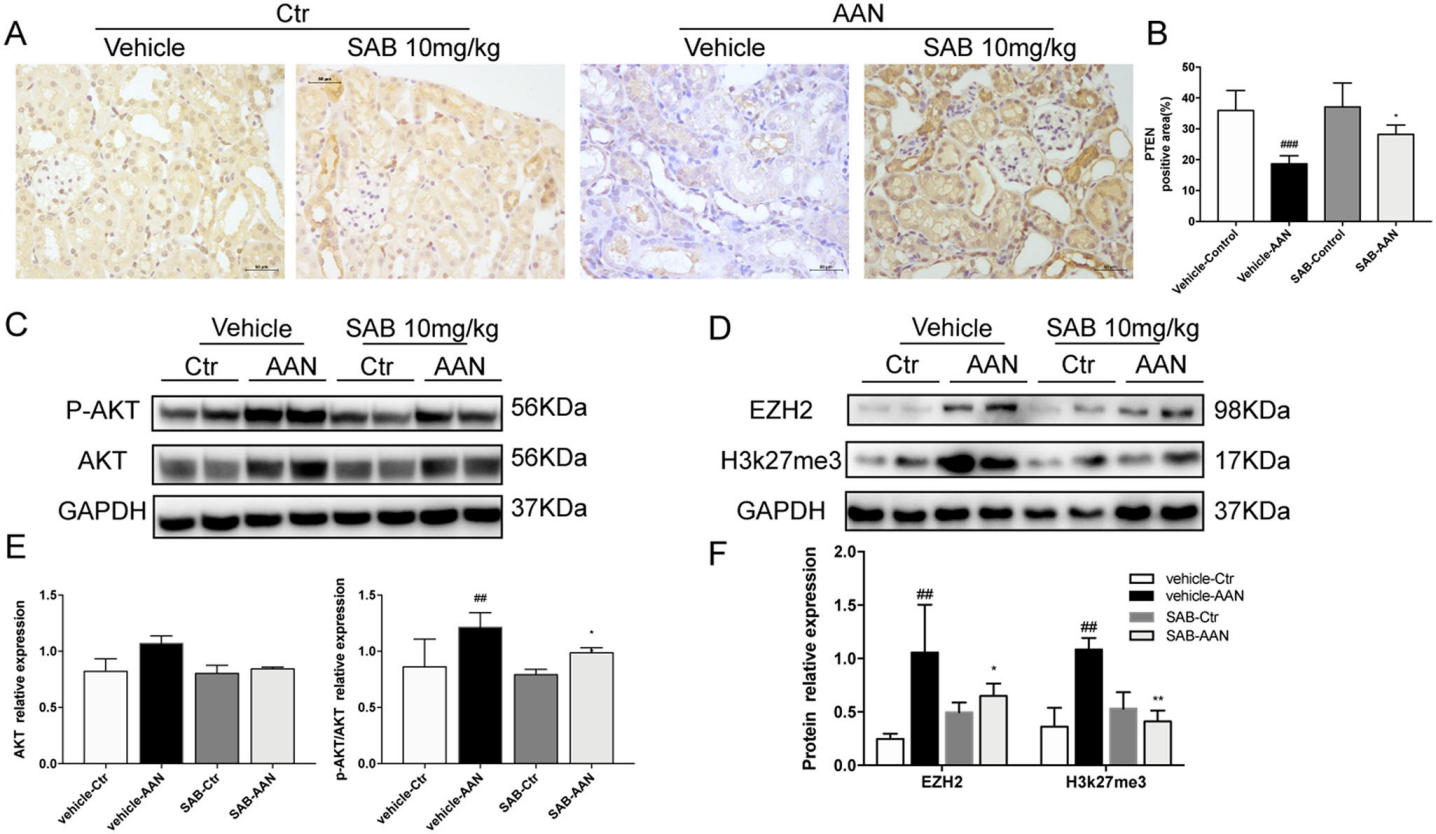
SAB alleviates renal fibrosis through inhibiting EZH2 and reversing the low level of PTEN and phosphorylation of AKT increase in AAN model kidneys. (A) Representative images of PTEN expression detected by IHC (magnification × 400, Bar = 50 μm). (B) Quantitative analysis of PTEN positive area (*n* = 6). (C) Immunoblot analysis of p-AKT and AKT. (D) Immunoblot analysis of EZH2 and H3k27me3. (E) The ratio of AKT to GAPDH protein and phosphorylated AKT to AKT protein was measured (*n* = 6). (F) The ratio of EZH2, H3k27me3 to GAPDH protein was measured (*n* = 6). ^##^*p* < 0.01 vs. vehicle-control; *p < 0.05 vs. vehicle-AAN; **p < 0.01 vs. vehicle-AAN.

The expression of PTEN and phosphorylation of Akt were examined in the kidney of the UUO and AAN mouse models. Immunohistochemical staining showed PTEN expression was suppressed in the kidney of both two models, and treatment with SAB reversed the expression of PTEN (Figures 3(A,B) and 4(A,B)). As shown in Figures 3(C,E) and 4(C,E), the expression of p-Akt in the model kidney tissues was markedly higher than in the normal group, the SAB dramatically reduced the phosphorylation of Akt.

### SAB inhibited TGF-β-induced fibrosis in NRK-49F cells

**Figure 5.**
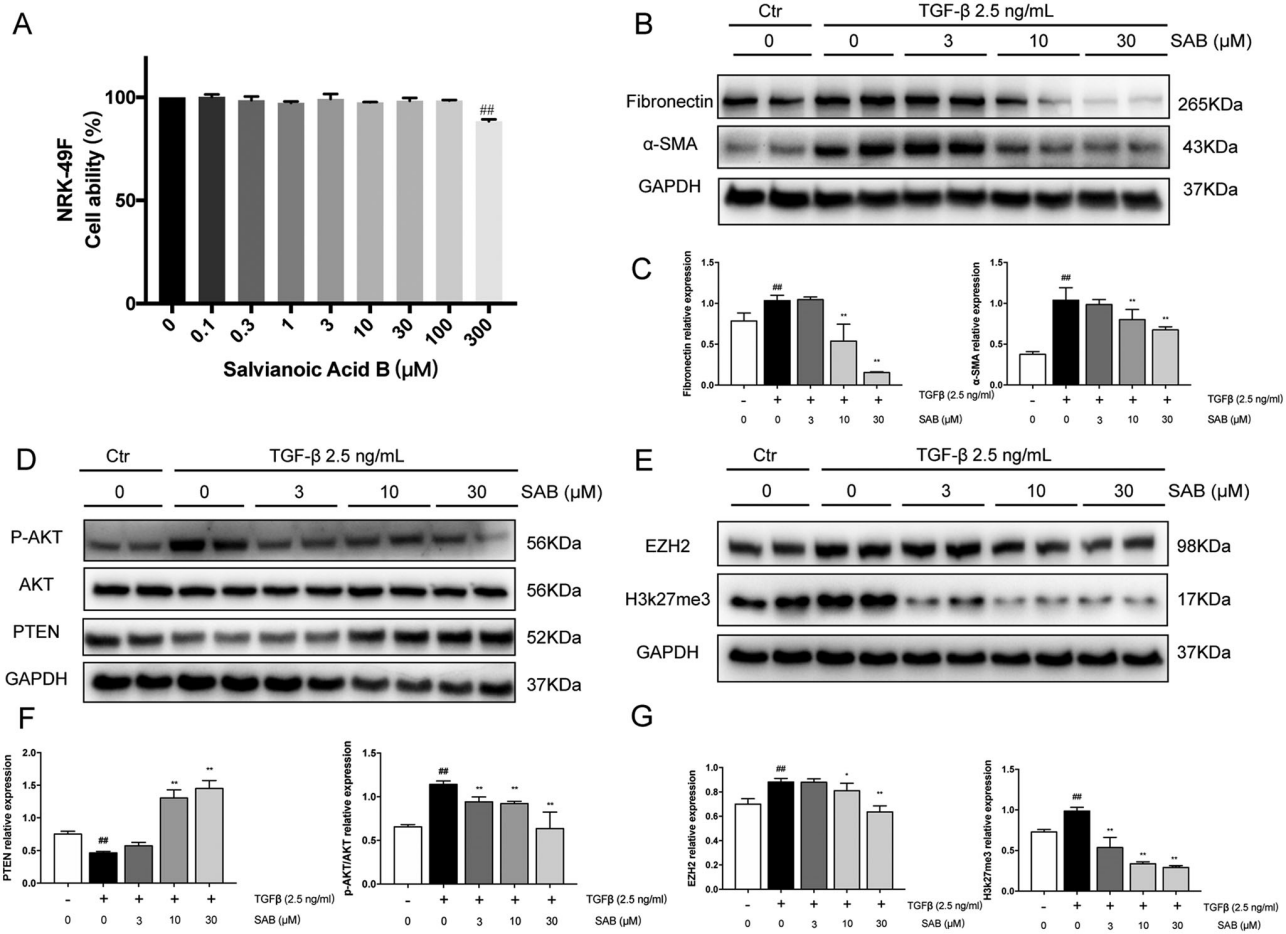
SAB protects against TGF-β induced fibrosis *in vitro*. (A) Effect of SAB on NRK-49F cell proliferation (*n* = 5), cells were treated for 24 h. (B, D, E) Immunoblot analysis of FN, α-SMA, PTEN, p-AKT, AKT, EZH2 and H3k27me3 expression. (C, F, G) The ratio of FN, α-SMA, PTEN, EZH2 and H3k27me3 to GAPDH protein and phosphorylated AKT to AKT protein was measured (*n* = 6). ^#^*p* < 0.05 vs. control group; ^##^*p* < 0.01 vs. control group; *p < 0.05 vs. model group; **p < 0.01 vs. model group.

Using rat renal interstitial fibroblast cells (NRK-49F) as models, we further confirmed the antifibrotic effect of SAB *in vitro*. Firstly, CCK-8 assay was performed to determine the safe concentrations of SAB on NRK-49F cells. As shown in Figure 5(A), compared to the control group, NRK-49F cells viability were decreased in highest concentration of 300 μM SAB. The expression of fibrotic markers such as FN and α-SMA were increased upon challenge with 2.5 ng/mL TGF-β for 24 h, SAB (3, 10, 30 μM) inhibited the expression of FN and α-SMA upon TGF-β stimulated NRK-49F cells (Figure 5(B,C)). Furthermore, challenge with TGF-β for 24 h decreased PTEN expression while increased p-Akt expression, SAB was effective in upregulating PTEN expression and downregulating the expression of p-Akt in NRK-49F cells (Figure 5(D,F)).

### SAB improved renal fibrosis through inhibiting EZH2 and h3k27me

To further explore the mechanism of SAB anti-fibrotic effect, the expression of EZH2 and h3K27me3 were analyzed. As show in Figures 3(D,F) and 4(D,F), the expression of EZH2 and h3K27me3 were increased in mice kidneys after UUO operation or after AAN injection, the SAB significantly reduced expression of EZH2 and H3k27me3. As shown in Figure 5(E,G), the expression of EZH2 and H3K27me3 were increased in TGF-β stimulated NRK-49F cells, whilst SAB inhibited the expression of EZH2 and H3K27me3 in a dosage dependent manner.

### SAB improved renal interstitial fibrosis by regulated PTEN/Akt pathway via EZH2

**Figure 6.**
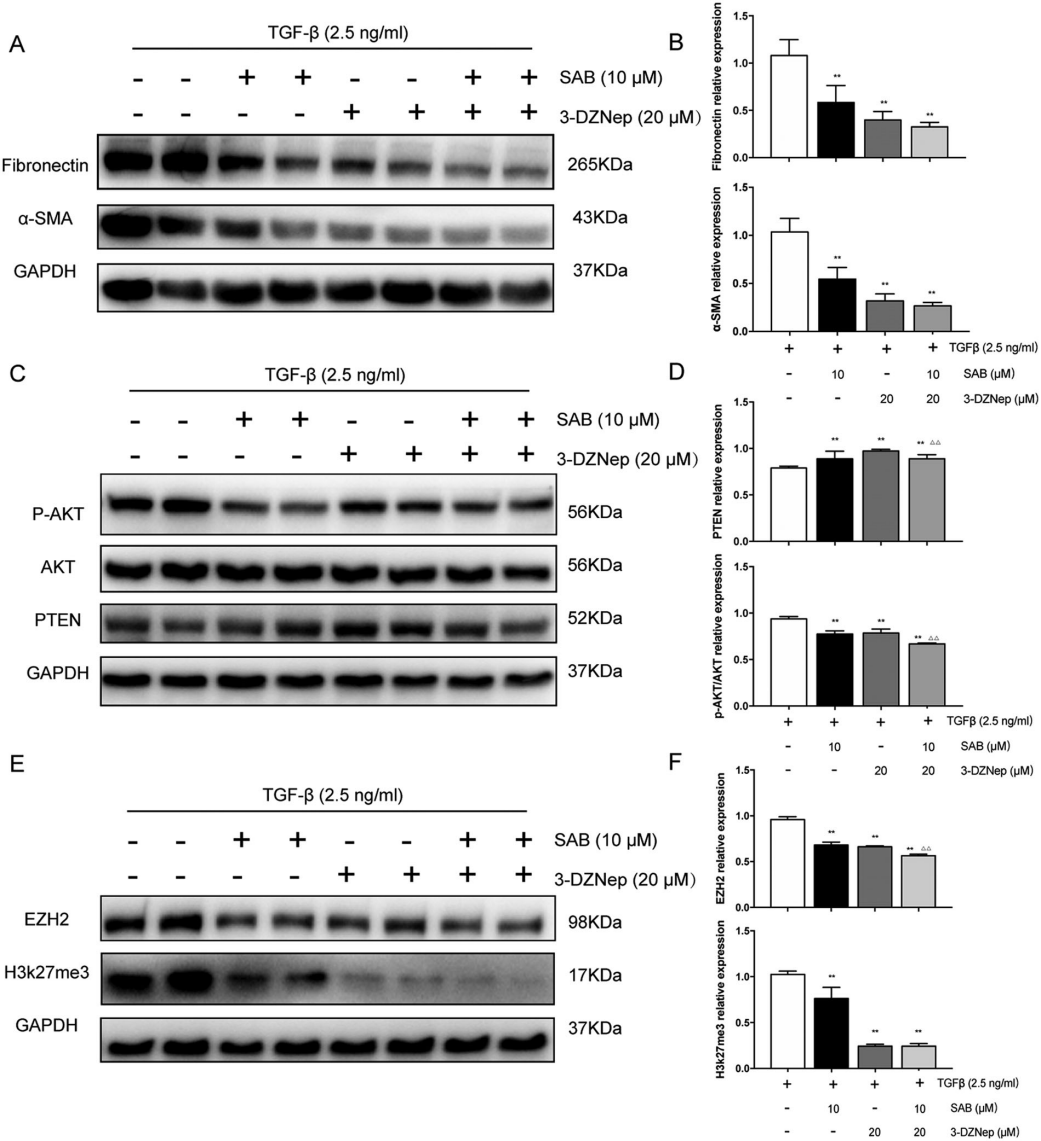
SAB protects against TGF-β induced fibrosis through inhibiting EZH2 and reversing the low level of PTEN and phosphorylation of Akt increase. (A, C, E) Immunoblot analysis of FN, α-SMA, PTEN, p-AKT, AKT, EZH2 and H3k27me3 expression. (B, D, F) The ratio of FN, α-SMA, PTEN, EZH2 and H3k27me3 to GAPDH protein and phosphorylated AKT to AKT protein was measured (*n* = 6). ^#^*p* < 0.05 versus control group, ^##^*p* < 0.01 vs. control group; *p < 0.05 vs. model group; **p < 0.01 vs. model group; ^△△^*p* < 0.01 vs. 3-DZNep group.

Figure 6 showed that SAB inhibited the expression of FN by 45.89%, α-SMA by 47.36% (Figure 6(A,B)), p-Akt/Akt by 17.28% and increased the expression of PTEN by 12.74% in the absence of 3-DZNep (Figure 6(C,D)), a potent inhibitor of EZH2, whilst SAB inhibited the expression of FN by 18.34%, α-SMA by 16.36%, or p-Akt/Akt by 14.69% even did not increase PTEN expression in the presence of 20 μM 3-DZNep. In parallel, SAB inhibited the expression of EZH2 by 29.02% and H3k27me3 by 25.70% without the addition of 3-DZNep, and inhibited the expression of EZH2 by 14.67% or even did not suppress H3k27me3 (Figure 6(E,F)).

## Discussion

SAB, one of the water-soluble components of salvianolic acids, which are main compounds found in Danshen, have good effects on some chronic fibrosis disease (Wu et al. 2019). Jiang et al. (2020) demonstrated that SAB could delay the progression of pulmonary fibrosis through inhibiting inflammatory response and regulating the fibrosis pathway. Moreover, several *in vitro* and *in vivo* studies implied that SAB was protective for renal tubular cells and interstitial cell. Hu et al. (2020) showed that SAB at a dose of 6.25 mg/kg decreased TGF-β and FGF-2 level and alleviated ECM components in the kidney of UUO mice model.

As a histone-lysine *N*-methyltransferase enzyme, EZH2 catalyses the addition of methyl groups to histone H3K27 and leads to the gene silencing (Duan et al. 2020). EZH2 plays a critical role in the development of renal fibrosis, which is expressed at a high level in both tubular epithelial cells and renal interstitial fibroblasts of human kidneys following CKD (Li et al. 2021). *In vitro,* pharmacological inhibition or siRNA mediated silencing of EZH2 reduced the activation of interstitial fibroblasts and epithelial cell EMT (Zhou et al. 2018). *In vivo*, treatment with 3-DZNep, the EZH2 inhibitor, alleviated the UUO-induced fibrosis (Zhou et al. 2016). The anti-fibrotic effect of EZH2 inhibition was related to the deactivation of TGF-β/Smad3, the regulation of PTEN/Akt signaling pathways to reduce renal interstitial ECM. EZH2 inhibition also reversed PTEN and repressed the transcription factors such as Snail-1 and Twist to regulate epithelial-mesenchymal transition in fibrotic kidneys. These results suggested that EZH2 was a promising therapeutic target for the treatment of renal fibrosis. Recent study showed that tanshinone I, a liposoluble constituent of *Salvia miltiorrhiza*, can bind directly to EZH2 and subsequent repression of the H3k27 methyltransferase activity for anti-leukemia therapies. But there was no research on whether SAB can inhibit EZH2 during renal fibrosis treatment (Huang et al. 2021).

In this study, we found that expression of EZH2 and H3K27me3 were upregulated in the kidneys of UUO and AAN models and TGF-β stimulated NRK-49F cells. Inhibition of EZH2 by 3-DZNep attenuated the anti-fibrotic effect and blocked the PTEN increasement as well as the p-Akt dephosphorylation. Furthermore, SAB suppressed the expression of EZH2 and its substrate h3k27me3 and thereby up-regulated protein expression of PTEN and decreased the ratio of p-Akt to Akt in two mice models and in NRK-49F cells. SAB significantly inhibited the expression of renal fibrosis-associated hallmarks and improved pathological structures as well as renal functions in UUO and AAN kidney, the murine model to investigate renal tubulointerstitial fibrosis. SAB significantly inhibited the expression of FN and α-SMA, p-AKT/AKT and increased the expression of PTEN. However, the effects of SAB on inhibiting the expression of FN, α-SMA, p-AKT/AKT was much weaker, even did not increase PTEN expression in the presence of 3-DZNep. These results suggested that SAB could protect renal tubulointerstitial fibrosis by inhibiting EZH2 to regulate PTEN/AKT signaling pathway.

## Conclusions

These findings suggested SAB treatment inhibited EZH2 and H3k27me3 through regulating PTEN/AKT signaling pathway, subsequently decreased the expression of FN and α-SMA to suppressed the renal fibrosis and improve renal function. However, therapeutic effects of SAB on renal fibrosis of CKD need further clinical trials to validate.
